# Modality-specific effects of threat on self-motion perception

**DOI:** 10.1186/s12915-024-01911-3

**Published:** 2024-05-23

**Authors:** Shira Hacohen-Brown, Eva Gilboa-Schechtman, Adam Zaidel

**Affiliations:** 1 Gonda Multidisciplinary Brain Research Center, Bar-Ilan University, 5290002 Ramat Gan, Israel; 2 Department of Psychology, Bar-Ilan University, 5290002 Ramat-Gan, Israel

**Keywords:** Vestibular, Visual, Multisensory, Trait anxiety, Attentional capture

## Abstract

**Background:**

Threat and individual differences in threat-processing bias perception of stimuli in the environment. Yet, their effect on perception of one’s own (body-based) self-motion in space is unknown. Here, we tested the effects of threat on self-motion perception using a multisensory motion simulator with concurrent threatening or neutral auditory stimuli.

**Results:**

Strikingly, threat had opposite effects on vestibular and visual self-motion perception, leading to overestimation of vestibular, but underestimation of visual self-motions. Trait anxiety tended to be associated with an enhanced effect of threat on estimates of self-motion for both modalities.

**Conclusions:**

Enhanced vestibular perception under threat might stem from shared neural substrates with emotional processing, whereas diminished visual self-motion perception may indicate that a threatening stimulus diverts attention away from optic flow integration. Thus, threat induces modality-specific biases in everyday experiences of self-motion.

## Background

Threat biases our perception of sensory events. People perceive threatening objects as being closer than non-threatening objects [1–3]. Threatening visual and auditory stimuli are perceived to approach us more rapidly than non-threatening stimuli [4–6]. Moreover, in both visual and auditory modalities, negatively valenced stimuli are perceived as longer in duration than positively valenced or neutral stimuli [7–9]. Thus, objects or events in the environment associated with threat are perceived with increased intensity.

Anxiety has been shown to influence the effects of threat on perception. Specifically, highly anxious individuals experience greater perceptual biases under perceived threat [10–13] and overemphasize the dangerousness and closeness of a threat [14]. Moreover, trait anxiety is associated with the predisposition to perceive a threatening stimulus as approaching more rapidly [15–17]. Similarly, trait anxiety is associated with overestimating the duration of threatening stimuli [18, 19]. Thus, anxiety seems to amplify the effects of threat on perception.

Most research on the effects of threat on perception per se, as well as on individual differences thereof, has focused on perception of events or objects in the environment and been limited mostly to the visual and auditory modalities. We know less about the effects of threat and anxiety on perception in other modalities, especially those related to interoception. Indeed, heightened anxiety is associated with altered perception of breathing [20]. It is also associated with increased heart rate estimates [21] and, in some conditions, greater interoceptive accuracy thereof [22], while in other conditions, reduced awareness of increased heartbeat [23, 24]. Standing on a raised platform in virtual reality (a threatening scenario) leads to changes in balance control and postural responses, such as leaning away from the platform edge [25]. This behavior is possibly mediated by increased arousal [26] and is reduced by repeated exposure [27–29]. Yet, less is known about the relationship between threat, anxiety, and other interoception-based information processing such as self-motion perception.

Veridical perception of self-motion (where one’s own body is moving in space) is crucial for everyday function—to maintain balance, navigate, forage, and escape danger. However, the effects of threat on self-motion perception are currently unknown. Recent neuroimaging studies have begun to shed light on this topic. Threatening self-motion stimuli (simulated rollercoaster) lead to increased activity in the left parieto-insular vestibular cortex (PIVC) and enhanced functional connectivity between the left PIVC and the right amygdala in individuals with high neuroticism [30]. However, whether self-motion perception (including dynamic vestibular stimulation) is altered by perceived threat, and whether trait anxiety modulates this effect, has not been tested. Understanding the way threat and trait anxiety affect self-motion perception is important for modeling subjective experience, given the continual motion we experience in our daily lives.

Self-motion perception in primates relies primarily on vestibular and visual cues [31–37]. Importantly, the vestibular and visual modalities are fundamentally different. The vestibular sense is evolutionarily old (found in all vertebrates, with invertebrate origins [38, 39]). It is one of the first senses to mature during development [40, 41] and controls low-level reflexes for balance and eye stability [42–45]. Vestibular function is also related to emotional processing, with comorbidities between vestibular impairments and anxiety disorders [46–55]. Highly anxious individuals are also more likely to experience hyper-sensitivity to vestibular stimuli [56, 57] and show greater dependence on vision than vestibular cues for postural control [58–61], possibly due to poor vestibular control or altered visual-vestibular integration [61–63].

While the vestibular sense is interoceptive in nature, dedicated to measuring one’s *own* orientation and motion in relation to the world, vision is primarily dedicated to sensing distant objects and events in the world, external to the self. Thus, visual perception of self-motion includes complex higher-level cognitive processing (i) to integrate multiple entities of optic flow across a large field of view [64–66] and (ii) to disambiguate optic flow resulting from self-motion, eye movements, and external objects moving independently in the environment [64, 67–71]. Hence, the influence of threat and trait anxiety on self-motion perception can be different for these distinct modalities.

In this study, we examined the effects of threat on self-motion perception, operationalized as concurrent threatening (versus neutral) auditory stimuli. Self-motion perception, in turn, was broken down into vestibular self-motion (without any visual component) and visually simulated self-motion (without any vestibular component). Examining the different modalities individually allowed us to differentiate modality-specific from general (supra-modal) effects of threat and trait anxiety on self-motion perception.

We made three predictions. First, consistent with previously found effects of threat on visual and auditory perception, we hypothesized that threat would intensify the experience of self-motion, for both modalities (*threat enhancement* hypothesis). Second, we expected vestibular-mediated self-motion perception to be more strongly affected by threat than visually-mediated self-motion perception (*vestibular enhancement* hypothesis; we still predicted threat enhancement for visual stimuli, but to a lesser degree). This hypothesis was based on (i) the notion that the primary (body-based) modality for self-motion perception is vestibular; (ii) visual optic flow alone (without vestibular) often takes longer (4 ~ 8 s) to elicit a strong feeling of vection [72, 73]; (iii) vestibular cues dominate in multisensory cortical area PIVC [74], and visual cues undergo recalibration to vestibular signals in multisensory ventral intraparietal (VIP) cortex [75–77]; and (iv) the aforementioned vestibular-emotional connection. Finally, consistent with previous literature on individual-differences in trait anxiety, we expected trait anxiety to be associated with a greater influence of threat on perception for both modalities (*trait anxiety enhancement* hypothesis).

## Results

**Fig. 1:**
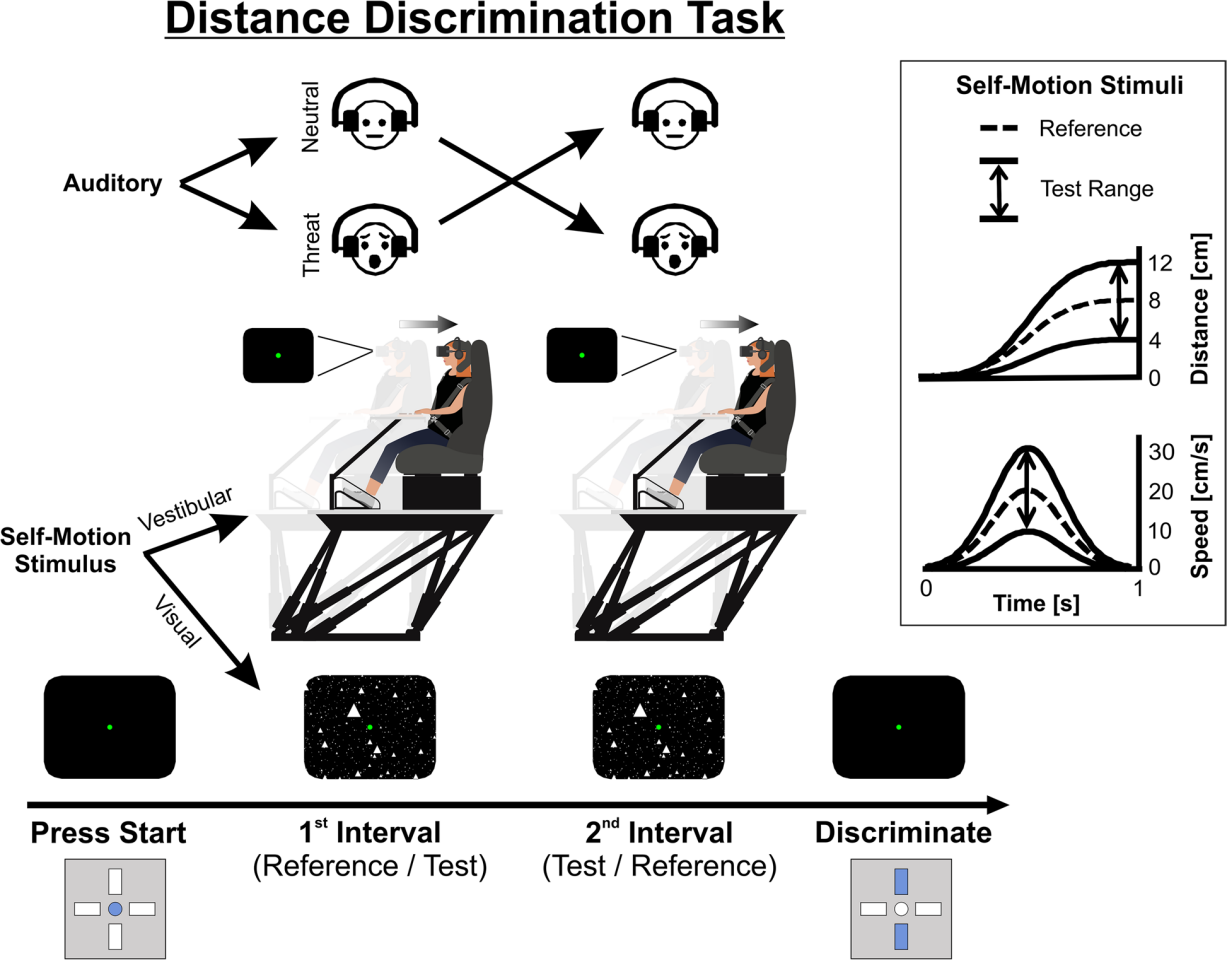
Setup and task. The sequence of a trial is presented along the arrow at the bottom of the figure. Each trial comprised two self-motion stimulus intervals that were either both vestibular or both visual. Motion platform schematics (center of the plot) depict two intervals of vestibular self-motion backward (semi-transparent shadings mark the starting position, before motion). Black screen schematics depict the visual scene (presented via a head mounted display). Visual self-motion was simulated through a 3D “star” field (white triangles). A green fixation point was always present. Face schematics (top) depict the two possible sequences of auditory stimuli: neutral → threat (or vice versa) played concurrently to the self-motion intervals. In the box on the right, self-motion stimulus profiles are presented: distance (top) and speed (bottom). Dashed curves reflect the reference motion stimulus (8 cm distance) and solid curves reflect the most extreme test motion stimuli (4 cm and 12 cm distance)

Forty-six healthy participants experienced self-motion stimuli in a motion simulator and performed a distance discrimination task (Fig. 1). On each trial, two self-motion intervals were presented from the same modality (either vestibular or visual). All self-motion stimuli were directed along a straight path backwards and lasted 1 s. One interval comprised a reference distance (8 cm), while the other comprised a test distance (range: 4–8 cm). Interval order (reference/test) was counterbalanced. Concurrent with each self-motion interval, a threatening or neutral auditory stimulus was played (also counterbalanced). After each trial, the participants reported which interval of self-motion covered a larger distance (see the “Methods” section for further details).

We found that threat affected self-motion perception in a modality-specific manner. Strikingly, threat had opposite effects on vestibular and visual self-motion perception, leading to overestimation of vestibular, but underestimation of visual self-motions. These results contradict the general threat enhancement hypothesis—only vestibular perception was enhanced (visual perception was diminished) under threat. But they support the vestibular enhancement hypothesis. In line with the trait anxiety enhancement hypothesis, a trend was seen between enhanced (supra-modal) self-motion perception under threat and trait anxiety scores; however, this result fell short of statistical significance.

### Opposite effects of threat on vestibular and visual self-motion perception

**Fig. 2:**
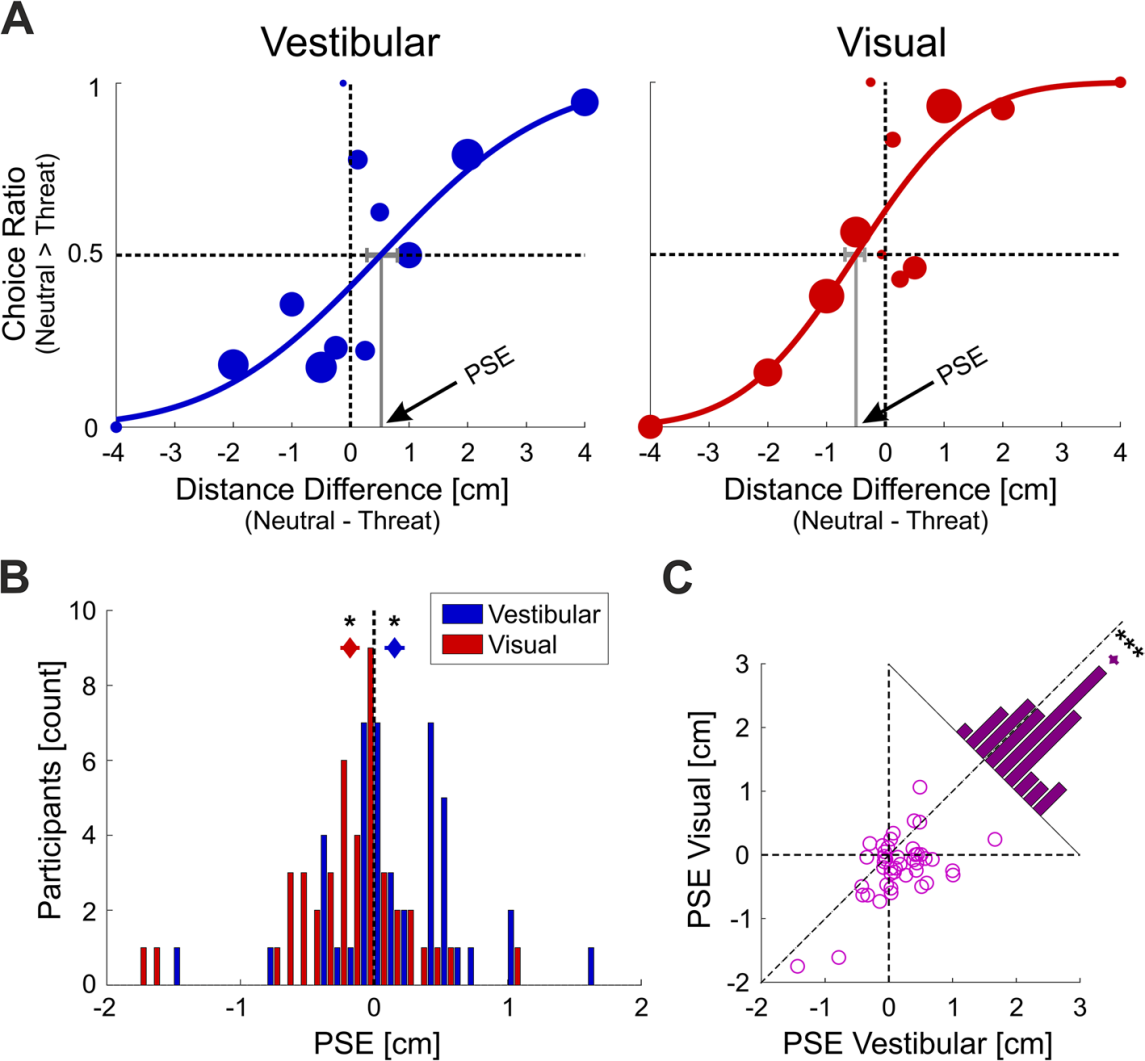
Modality-specific effects of threat. **A** Vestibular (left panel, blue) and visual (right panel, red) psychometric curves are presented for an example participant. The filled circle data points represent the ratio of choices that the distance during the interval with neutral audio was larger than that with threatening audio, as a function of the actual distance difference. Marker sizes reflect the number of trials per data point. Solid blue and red lines present psychometric fits to the data using cumulative Gaussian distribution functions. The point of subjective equality (PSE, marked by arrows and vertical solid gray lines) is the *x*-value at which the psychometric function equals 0.5. The horizontal gray lines mark PSE ± SEM (68% confidence intervals). Horizontal black dashed lines mark *y* = 0.5. Vertical black dashed lines mark *x* = 0 (where both intervals covered the same distance). **B** Distribution of vestibular (blue) and visual (red) PSEs (*n* = 44). The vertical black dashed line (PSE = 0) marks no effect of threat. Diamond markers and error bars above the histograms mark the modality-specific mean ± SEM values. **C** Scatter of visual vs. vestibular PSEs (*n* = 44). Each data point represents one participant. The histogram on the diagonal depicts the distribution of ΔPSE (PSE_vestibular_ − PSE_visual_) across participants. The diamond marker and error bars above the histogram mark the mean ± SEM. *p < 0.05, ***p < 0.001

Vestibular and visual psychometric curves for an example participant are presented in Fig. 2A (left and right panels, respectively). The point of subjective equality (PSE; marked by arrows) quantifies the effect of threat on self-motion perception. In the vestibular condition (left panel), a positive PSE is seen. This indicates that a smaller distance in the threat condition was perceived equal in magnitude to a larger distance in the neutral condition. Thus, threat increased the perceived magnitude of vestibular self-motion. By contrast, in the visual condition (right panel), threat enhancement was not seen. To the contrary, an opposite effect (negative PSE) is seen. The negative PSE indicates that a larger distance in the threat condition was perceived equal in magnitude to a smaller distance in the neutral condition. Thus, threat reduced the perceived magnitude of visual self-motion.

At the group level, when ignoring modality (using the average vestibular and visual PSE, per participant), there was no significant effect of threat on self-motion perception (mean ± SEM: − 0.013 ± 0.43 cm; *t*_(43)_ = − 0.2, *p* = 0.58, Cohen’s* d* = − 0.03; one-tailed* t*-test). Thus, a general *threat enhancement* hypothesis, which threat magnifies self-motion perception irrespective of modality, is not supported by the results. Further inspection of the results for each modality, separately, reveals that only vestibular (but not visual) self-motion perception was enhanced by threat. In the vestibular condition, participants significantly overestimated distances of self-motion with threat compared to neutral (PSE mean ± SEM = 0.15 ± 0.08 cm; *t*_(43)_ = 2.06, *p* = 0.023, Cohen’s* d* = 0.31; one-tailed *t*-test), whereas in the visual condition, participants did not overestimate distances of self-motion with threat compared to neutral (PSE mean ± SEM: − 0.18 ± 0.07 cm; *t*_(43)_ = − 2.50, *p* = 0.99, Cohen’s* d* = − 0.38; one-tailed *t*-test).

The distribution of vestibular and visual PSEs across participants is presented in Fig. 2B (blue and red histograms, respectively). Post hoc inspection of these data revealed strong modality-specific differences. While the vestibular PSEs were in line with the threat enhancement hypothesis, the visual PSEs indicated an opposite effect—underestimation of self-motion distances under threat. Accordingly, we performed a two-tailed *t*-test (post hoc) and indeed observed significantly diminished perception of visual self-perception under threat (*p* = 0.016)*.* Lastly, non-parametric statistical testing confirmed that the vestibular and visual results were not driven by outliers (*p* = 0.020 for vestibular, and *p* = 0.006 for visual, two-tailed Wilcoxon signed-rank test).

A paired comparison exposes the difference between modalities more robustly (Fig. 2C). The scatter plot of visual vs. vestibular PSEs shows that the majority of individuals have larger vestibular vs. visual PSEs (33 out of 44 data points lie below the diagonal line of equality). And the difference (PSE_vestibular_ − PSE_visual_, histogram) was significantly positive (mean ± SEM = 0.33 ± 0.07 cm; *t*_(43)_ = 4.80, *p* = 9.68·10^−6^ Cohen’s* d* = 0.72; one-tailed *t*-test). Thus, the data strongly support the *vestibular enhancement* hypothesis. Interestingly, despite the modality differences, vestibular and visual PSEs were significantly correlated (*r*_(42)_ = 0.55, *p* = 9.9·10^−5^). This suggests that individuals have idiosyncratic, supra-modal, baselines for the effect of threat, upon which the modality differences ride.

### Trend between enhanced self-motion perception under threat and trait anxiety

**Fig. 3:**
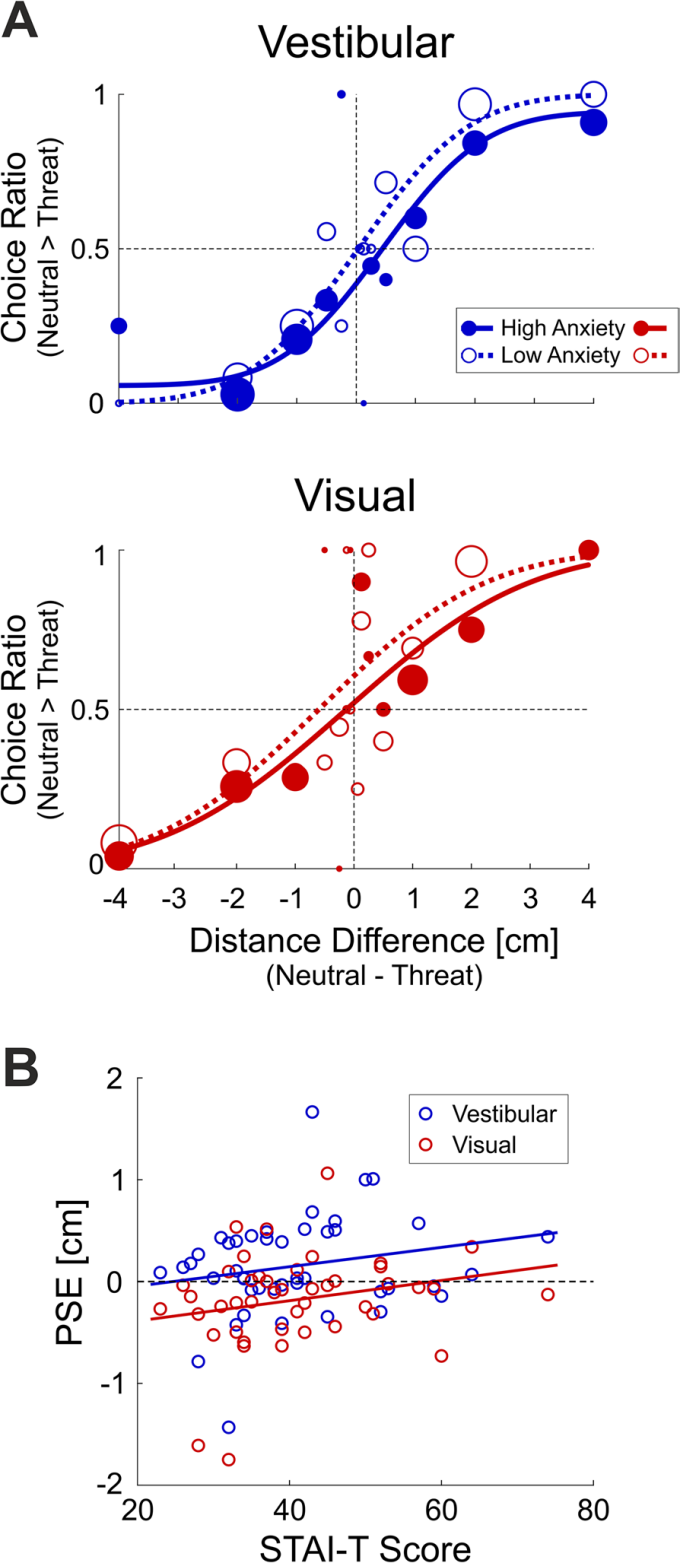
Individual differences in the effects of threat on self-motion perception. **A** Vestibular (top panel, blue) and visual (bottom panel, red) psychometric curves of two participants—one with high trait anxiety (STAI-T score 74; filled circles and solid lines) and one with low trait anxiety (STAI-T score 34; open circles and dashed lines). **B** Vestibular and visual PSEs (blue and red, respectively) as a function of STAI-T scores (*n* = 44), with regression lines per modality

Next, we tested whether trait anxiety levels (quantified by the trait component of the State-Trait Anxiety Inventory, STAI-T) explain some of the variance between individuals in terms of their baseline effect of threat on self-motion perception. Vestibular and visual psychometric curves are presented in Fig. 3A (top and bottom panels, respectively) for two example participants—one with high trait anxiety (STAI-T score 74; filled circle markers and solid lines) and one with low trait anxiety (STAI-T score 34; open circle markers and dashed lines). For both modalities, the high trait anxiety participant’s psychometric curves lie to the right of the low trait anxiety participants. Thus, the high trait anxiety participant’s PSEs are more positive, demonstrating larger overestimation of self-motion under threat. The modality effect (described in the previous section) can be also seen in these plots: the vestibular PSEs are more positive compared to the visual PSEs for both participants. This suggests that the two effects are superimposed (additive)—a modality-specific effect of threat and a supra-modal effect of trait anxiety.

To investigate the effects of trait anxiety, we performed a repeated measures ANCOVA (with modality as the repeated measure factor, and the standardized STAI-T scores as a covariate). Firstly, this analysis confirmed a significant main effect for modality, in line with the previous section. Vestibular PSEs were significantly larger than visual PSEs (*F*_(1,42)_ = 22.52, *p* = 2.42·10^−5^, ${\eta }^{2}$ = 0.107, ${\eta }_{p}^{2}$ = 0.35). Regarding trait anxiety, participants with higher STAI-T scores tended to have larger PSEs (overestimated self-motion distances under threat, Fig. 3B). However, this did not reach significance (*F*_(1,42)_ = 2.76, *p* = 0.10). No interaction between modality and trait anxiety was seen (*F*_(1,42)_ = 0.004, *p* = 0.95, ${\eta }^{2}$ = 2.09·10^−5^, ${\eta }_{p}^{2}$ = 1.05·10^−4^). Near parallel regression lines for vestibular and visual cues are in line with a supra-modal influence of trait anxiety (additive to the modality effect).

### Comparable results when using logarithmic distance differences

To test for other possible differences between the reference and test intervals, we performed a two-way repeated measures ANOVA of the following: stimulus (visual/vestibular) × condition (test/reference). The difference between stimuli remained highly significant (*F*_(1,43)_ = 23.69, *p* = 2·10^−5^, ${\eta }^{2}$ = 0.137, ${\eta }_{p}^{2}$ = 0.355), in accordance with vestibular enhancement. The results also showed a significant effect of interval (*F*_(1,43)_ = 9.35, *p* = 0.0038, ${\eta }^{2}$ = 0.075, ${\eta }_{p}^{2}$ = 0.179), such that, on average (across both cues and threat/neutral stimuli on the test/reference interval), the reference interval was judged larger than the mean test intervals. We understand this in terms of the range of test distances (4 cm to 12 cm) vs. the reference distance (8 cm). Although the arithmetic differences between the test and reference distances had equal magnitudes symmetrically, the scaling could be logarithmic, rather than linear. For example, the difference between 8 cm vs. 4 cm could be perceptually larger than 12 cm vs. 8 cm (although both differ by 4 cm, the former has a 2:1 ratio; while the latter has a 3:2 ratio). Accordingly, the reference interval would be judged larger, on average, than the mean test interval. Because the data were counterbalanced (the threat and neutral stimuli were given on both the test and reference intervals, an equal amount, for each participant), this would not affect the results and conclusions of this study. However, to further validate this, we reran all the analyses using logarithmic distance differences (rather than arithmetic differences) for the *x*-values of the psychometric functions. All the results remained qualitatively identical.

## Discussion

In this study, we examined the effects of threat and trait anxiety on perception of self-motion. We did not find support for a general (supra-modal) influence of threat on self-motion perception (*threat enhancement* hypothesis). Rather, we found differential effects of threat on the vestibular and visual modalities: while threat intensified perception of vestibular self-motion, it diminished perception of visual self-motion. These results support the *vestibular enhancement* hypothesis and reveal an unexpected effect of threat on visual self-motion perception. In line with the *trait anxiety enhancement* hypothesis, a person’s level of trait anxiety tended to increase the perceived intensity of self-motion under threat. However, this trend did not reach statistical significance.

### Modality-specific effects of threat on self-motion perception

Differential effects of threat for the vestibular and visual modalities may be related to the intrinsically distinct nature of these modalities. The vestibular sense is “body-based” and dedicated to detecting one’s own motion in space. Also, additional body-based cues (somatosensory and proprioceptive) may have contributed to a richer perceptual experience of self-motion in the vestibular condition [42, 78–80]. By contrast, the human visual system is dedicated primarily to perceiving external objects and events in the environment. Without concurrent vestibular cues, a strong feeling of vection from visual stimuli might only be elicited from longer durations (> 4 s) of optic flow [72, 73, 81]. It is therefore possible that the experience of self-motion with visual cues alone was weaker (less embodied), compared to the rich, body-based, experience in the vestibular condition. This notion suggests that concurrent vestibular and visual self-motion perception would be intensified by threat. Alternatively, it is also possible that the opposite effects of threat for visual and vestibular cues could cancel out when presented together. Future research with combined (vestibular-visual) stimuli can answer this question.

Different neural processing of vestibular and visual self-motion information may account for the differential effects of threat on perception in the vestibular and visual modalities. Vestibular processing is inherently multisensory and relies on broad subcortical, cerebellar, and cortical brain networks [42, 82–85]. Brain areas with strong vestibular signals are interconnected with regions that process emotion. Multisensory cortical area PIVC has dominant vestibular responses (with little tuning to visual optic flow [74]) and is directly connected to the amygdala [86]. Also, individuals with high neuroticism show increased functional connectivity between the left PIVC and the right amygdala [30]. PIVC and the amygdala are also both connected to the anterior insula, which is closely associated with emotional processing [30, 87–89]. However, with vestibular signals broadly distributed across the brain, neuronal connections to emotional processing likely manifest at multiple levels.

Subcortically, the parabrachial nuclei have two-way connections with both the vestibular nuclei [46, 62, 90] and the amygdaloid nuclei [62, 91, 92]. Additionally, neuroticism is associated with increased activity in the pons (which contains the parabrachial nuclei) and increased connectivity between the pons and the amygdala [93]. Accordingly, the effect of threat on vestibular motion perception may be explained by functional connectivity between areas that process vestibular and emotional responses. For example, increased activity in the amygdaloid nuclei from threat or trait anxiety [94] might heighten sensitivity in the parabrachial and vestibular nuclei and thereby intensify vestibular perception of self-motion.

Visual self-motion perception requires high-level cortical mechanisms to integrate information across the visual field [65, 66] and to dissociate self-motion from object motion in the environment [67, 70, 71, 95, 96]. These functions rely predominantly on extra-striate visual cortex, most notably the dorsal medial superior temporal area (MSTd) [97–100]. MSTd also has vestibular signals, but to a lesser degree, and neuronal perturbation primarily influences visual (vs. vestibular) self-motion perception [98, 101, 102]. A threatening stimulus may attract attention (attentional capture) away from the process of visual self-motion perception, resulting in reduced estimates of visual self-motion. Indeed, concurrent performance of another cognitive task diverts attention away from optic flow processing [103]. Accordingly, concurrent threat may detract attention away specifically from visual self-motion processing, which relies heavily on high-level cortical processing, and not vestibular motion processing, which is more distributed across the brain, with strong low-level responses to self-motion [84, 104].

Altered perception of self-motion intensity under threat might be mediated in part by changes in time perception [105], which are affected by arousal levels and attentional resources [106, 107]. First, increased arousal can lead to a perceived lengthening of time [108]. Accordingly, if individuals estimate vestibular distances by integrating motion over time, and they overestimate the duration of the vestibular stimulus in the threat condition, increased estimates of distance may follow. By contrast, attentional capture (by an irrelevant distractor) or high cognitive load can lead to perceived shortening of time [107, 109–112] and distance traveled [105]. Thus, if participants underestimate visual stimulus duration in the threat condition due to diverted attention away from the integration of visual optic flow, this could lead participants to underestimate the distance traveled. This suggestion provides a testable hypothesis: the effect of threat on stimulus time perception would be different for the two modalities.

### Trait anxiety and self-motion perception

A trend was seen between trait anxiety and the influence of threat on self-motion perception, supra-modally. However, this did not reach statistical significance. Thus, future research with a larger cohort and/or clinical anxiety group is needed to confirm this observation. Anxiety could influence self-motion perception via modulatory projections from the amygdala to brain areas that process self-motion [113–116]. Individuals who suffer from anxiety have stronger connectivity between the amygdala and parietal regions [117–119]. This could amplify the influence of threat on self-motion perception. This idea is in line with previous findings that perceptual biases under threat are generally enhanced by trait anxiety [10–13].

### Limitations and future directions

We note here several limitations of this study. The auditory stimuli for threat manipulation (threatening vs. neutral conditions) differed significantly in terms of valence and arousal. Clearly, valence and arousal are inherently linked: positive and negative valence are both accompanied by high arousal, while neutral valence is accompanied by low arousal [120]. Therefore, when comparing conditions with threatening (negative) valence vs. neutral valence, arousal is a confounding factor. Also, auditory stimulation in itself can change estimates of self-motion [121]. We did not measure any effects (irrespective of threat) that the auditory cues had on self-motion, and relied on the paired (two-interval) design, for control. Thus, future work is required to study these aspects.

Stimulus distance and speed are linearly related (when stimulus duration is constant). Thus, although participants were instructed to judge the distance traveled, they could use speed as a proxy. Thus, we cannot isolate which motion parameter (distance or speed) is perceived differently under threat. Additional research designed to dissociate motion parameters is required to tease apart the specific effects of threat on distance, speed, and time perception. Also, our study examined only linear backward motions. Future research should test whether the effects found here generalize to forward, sideward, and vertical motions as well as rotational motions. Regarding the visual stimuli, a feeling of vection might be lacking for short duration stimuli, and perception of distance could be compressed in virtual reality. Thus, future research should investigate whether threat leads to enhanced multisensory (combined visual-vestibular) self-motion perception or whether the opposite effects of threat on visual and vestibular perception observed here cancel out.

Finally, participants in this study were likely to experience a compromised sense of agency while performing the task: although they controlled the initiation of motion, they lacked control over its magnitude and direction. The participants could not stop the motion, slow it down, speed it up, or change its direction. Also, vestibular motions could be more susceptible (vs. visual) to effects of threat. Therefore, further research is required to test the generalizability of our findings to situations characterized by varying senses of agency. Testing the intensity of perceived self-motion in drivers compared to passengers who experience different senses of agency but receive similar sensory inputs may help us understand how agency affects self-motion perception under threat. Future work should also investigate possible implications of these findings in real-world situations of self-motion under threat, such as car accidents.

## Conclusions

This study explored how threat impacts one’s perception of self-motion in space. We found modality-specific effects. Under threat, vestibular mediated self-motions were overestimated, whereas visually mediated self-motions were underestimated. A (non-significant) trend was seen between trait anxiety and increased perceived self-motion under threat, for both modalities.

## Methods

### Participants

We tested 46 healthy participants in this study (27 females; mean age ± SD = 25.2 ± 2.9 years, range: 20–34). This sample size was commensurate with previous studies testing similar effects [122, 123]. All participants performed both self-motion conditions (visual and vestibular) on the same day, except one who performed the experiment on two different days. All participants had normal hearing and normal or corrected-to-normal vision and reported no history of psychiatric or neurological disorders. Right before performing the experiment, participants completed the State-Trait Anxiety Inventory, trait component (STAI-T) questionnaire [124]. This was used to assess the participants’ levels of trait anxiety (similar to previous studies; [19, 20, 57, 123, 125–128] to test whether the effects of threat on perception correlate with an individual’s trait anxiety. The mean STAI-T score ± SD across the cohort was 40.88 ± 10.97.

### Experimental setup

Participants were seated comfortably in a car seat that was mounted on a six-degrees-of-freedom motion platform (MB-E-6DOF/12/1000, Moog Inc.). They were restrained safely with a 4-point harness, and their heads were supported by a head support with lateral arms to limit head movements (Black bear, Matrix Seating Ltd.). Participants wore a virtual reality head-mounted display (HMD, Oculus Rift CV1) and noise-canceling headphones (Sony WH-1000XM3). A green fixation point was presented in the HMD and remained at a fixed distance (66 cm) in front of the participant throughout the experiment (i.e., it moved with the participant during self-motion stimuli). The participants were instructed to keep their heads straight and still and to focus on the fixation point throughout the experiment. The participants initiated trials and reported their selections via a response box (Cedrus RB-540).

### Self-motion stimuli

Stimuli comprised self-motions in a backward direction. Backward (rather than forward) self-motions were used because contracting optic flow (simulating backward self-motion) elicits a stronger feeling of vection than expanding optic flow (simulating forward self-motion) [73]. We also reasoned that backward self-motions might be more frightening and therefore more susceptible to the effects of threat and trait anxiety than forward motions. The vestibular stimuli comprised backward motions of the motion platform, upon which the participant was seated, in darkness (no visual cues apart from the fixation point). Although additional non-vestibular (e.g., somatosensory and proprioceptive) cues may also be used in this condition, we call this condition “vestibular” because performance relies heavily on an intact vestibular sense (vestibular lesion severely damages this ability [33, 129]. But it most likely comprises a mixture of body-based cues.

The visual stimuli (optic flow) were generated using OpenGL and presented binocularly in the HMD, with a field of view that spanned 88° horizontally and 90° vertically. The visual stimuli simulated backward self-motion through a 3D field of “stars.” The star field was 130 cm wide, 130 cm tall, and 100 cm deep and centered at 66 cm in front of the participant before motion onset. Star density was 0.00125/cm^3^, with each star being a 0.5 cm wide × 0.5 cm tall white triangle. A clipping plane was set at 5 cm in front of the participants’ eyes to prevent stars from being too close and large. All self-motion stimuli (vestibular and visual) were directed along a straight path backwards, and followed a Gaussian velocity motion profile, with 1 s duration (Fig. 1, box inset).

### Auditory stimuli

Threat during the self-motion stimuli was manipulated by concurrently playing auditory stimuli with threatening or neutral sounds. The auditory stimuli were taken from the International Affective Digitized Sounds (IADS) standardized database for affective auditory stimuli [130]. We used the expanded version [131] that grades the stimuli not only by factors of arousal and valence but also based on the emotions they provoke (e.g., fear, happiness, and sadness). Five threatening and five neutral auditory stimuli were selected. The threatening stimuli consisted of screams, while the neutral stimuli consisted of human chatter and other naturalistic sounds, such as a lawnmower. A representative 1-s section was custom selected from each of the selected IADS stimuli (which were originally 6 s long) to match the duration of the self-motion stimuli, and the peak amplitude was normalized such that all stimuli had the same maximum volume (after shortening), using the Audacity audio-editing software (Version 2.3.3).

The selected threatening and neutral stimulus sets differed significantly (based on the ratings from Yang et al. [131] on a 9-point scale) in fear-induction propensities (mean ± SD fear score was 7.36 ± 0.35 for threatening and 2.50 ± 0.68 for neutral; *t*_(8)_ = − 14.3, *p* = 5.5·10^−7^, Cohen’s* d* = − 9.1; *t*-test), valence (mean ± SD valence score was 1.84 ± 0.50 for threatening and 4.74 ± 0.90 for neutral; *t*_(8)_ = 6.3, *p* = 2.3·10^−4^, Cohen’s* d* = 4.0; *t*-test), and arousal (mean ± SD arousal score was 7.34 ± 0.43 for threatening and 6.12 ± 0.47 for neutral;* t*_(8)_ = − 4.3, *p* = 0.003, Cohen’s* d* = − 2.7; *t*-test). Similarly, the selected threatening stimuli differed significantly from the overall database in all three measures (valence, arousal, and fear), while the selected neutral stimuli did not differ in valence or arousal, but did have marginally lower fear scores, in comparison to the overall database.

### Distance discrimination task

Participants performed a two-interval distance discrimination task (Fig. 1). On each trial, they experienced two self-motion stimulus intervals and were required to report which one covered a larger distance. Only one modality was tested per trial—i.e., both the self-motion stimuli in a trial were either vestibular or visual. Each trial comprised one “reference” interval, of constant magnitude (8 cm distance), and one “test” interval, of varying magnitude (4–12 cm distance). The order of intervals (reference and test) within a trial was randomized. Each interval (for both vestibular and visual stimuli) lasted 1 s, and they were separated by a 1-s period with no stimulus. The second interval began where the first interval had ended, and the motion platform returned to the origin only after the trial was completed.

Trial difficulty was controlled by the difference between the test and reference distances (*D*). Large values of |*D*| (absolute value of the difference) reflect easy discriminations, whereas small values reflect difficult discriminations. Each block started with the largest absolute difference (|*D*|= 4 cm, i.e., test distances were 8 ± 4 = 4 and 12 cm). This was then reduced (discriminations became more difficult) according to a staircase procedure [132], as follows: after a correct response, |*D*| was decreased by a factor of 2 (i.e., the task became more difficult) with *p* = 0.3 (and remained unchanged with *p* = 0.7). After an incorrect response, |*D*| was increased by a factor of 2 (i.e., the task became easier) with *p* = 0.8 (and remained unchanged with *p* = 0.2). This staircase rule converges to a rate of ~ 73% correct choices [36, 133]. Thus, the possible values for *D* were as follows: ± 4, ± 2, ± 1, ± 0.5, ± 0.25, ± 0.125, ± 0.0625, etc. (there was no lower bound on |*D*|). The sign of *D* (whether the test distance was larger or smaller than the reference distance) was chosen randomly on each trial, with *p* = 0.5. Motion profiles are presented in Fig. 1 (box inset, distance and speed in the upper and lower subplots, respectively) for the two most extreme test stimulus trajectories (*D* = ± 4 cm; solid lines) and the reference stimulus (dashed lines).

The experiment comprised two vestibular blocks and two visual blocks. Each block contained 80 trials (i.e., 160 trials per modality, 320 trials in total). For each trial, a threatening and a neutral audio stimulus was randomly selected from their respective sets (with uniform distribution). The coupling of auditory condition (threat vs. neutral) to stimulus interval (reference vs. test) was counterbalanced across blocks. In one block, the neutral auditory stimuli were played during the reference intervals, and the threat stimuli were played during the test intervals. Reciprocally, in the other block, the neutral auditory stimuli were played during the test intervals, and the threatening stimuli were played during the reference intervals. Intervals, and thus threat conditions, were in random order in a trial. The order of blocks was counterbalanced across participants.

Participants were instructed to report whether the first or the second interval covered a larger distance (two-alternative forced-choice) by pressing the upper or lower button, respectively, on the response box (Fig. 1, bottom right). The following timing signals (audio beeps, unrelated to the audio affective cues) were given during the trial: (1) at the beginning of each trial, a beep signaled that the system was ready for the participant to initiate a trial. Participants could then initiate the trial by pressing the center button on the response box (Fig. 1, bottom left). (2) Following the discrimination decision (which was only possible after the second interval had ended), a different beep indicated that the choice was registered. (3) If no selection was made within a 2 s window after the second interval had ended, an error beep (timeout) was triggered. Participants were instructed to avoid this. Participants were given a few practice trials before starting the experiment to make sure that the instructions were well understood and that they pressed the buttons reliably. In this practice stage only, participants received verbal feedback from the experimenter regarding the correctness of their choices. No such feedback was provided thereafter during the actual experiment.

### Data analysis

Data analysis was performed with custom software using MATLAB R2018a (MathWorks). For each participant and stimulus modality (visual and vestibular), the data from the two blocks were pooled. If no response was recorded on a trial due to timeout, it was excluded from the analysis. First, we calculated the proportion of “neutral > threat” choices (i.e., the rate at which the participant chose that the distance coupled with the neutral audio was greater than the distance coupled with the threatening audio) as a function of the actual difference between those distances (neutral − threat; the same magnitude as *D*, but with sign set according to the threat condition rather than interval type). Psychometric curves were then generated by fitting the data with cumulative Gaussian distribution functions, using the psignifit toolbox (version 4) for MATLAB [134]. The goodness-of-fit of the psychometric functions was evaluated using pseudo-*R*^*2*^ [135]. Participants with pseudo-*R*^*2*^ values lower than 0.5 for either one of their psychometric functions (vestibular or visual) were removed from further analysis. This excluded two participants (leaving 44 for further analysis). The mean ± SD pseudo-*R*^*2*^ for the remaining psychometric functions was 0.85 ± 0.07 for vestibular and 0.84 ± 0.09 for visual.

The point of subjective equality (PSE) was defined by the mean of the fitted cumulative Gaussian psychometric function. The PSE quantifies the difference between the neutral and threat distances that would be judged by the participant to be equal. It comprised the primary dependent variable in this study. PSE = 0 indicates that the participant’s distance estimates were not affected by threat. Positive PSE values indicate that distances in the threat condition were perceived as larger than the distances in the neutral condition; conversely, negative PSE values indicate that distances in the threat condition were perceived as smaller than the distances in the neutral condition. Lastly, we compared discriminations of reference vs. test intervals irrespective of threat/neutral coupling and redid all the analyses in this manuscript using logarithmic (rather than arithmetic) distance differences for the psychometric fits (all the results and statistics remained qualitatively identical).

### Statistical analysis

Statistical analyses were performed using MATLAB and JASP (version 0.14.0.0), with PSE as the dependent variable. In this experimental design (two-interval distance comparison), the dependent variable (PSE) comprises only one measurement, which reflects the difference between the threat and neutral distance estimates. Thus, the null hypothesis for PSE was *μ* = 0. A two-way ANOVA of condition (neutral/threat) × stimulus (visual/vestibular) could not be performed (this would require two separate distance estimates for the threat and neutral conditions, not available for this type of within-subject design). For this same reason, we could not compare perceptual sensitivity between threat and neutral conditions, which would require separate psychometric curves to measure separate slopes (or thresholds, *σ*), per condition.

The *threat enhancement* hypothesis was tested by comparing the modality-independent PSEs (i.e., average of the visual and vestibular PSEs, per participant) to zero. This was done using a one-tailed *t*-test, in line with the a priori hypothesis that threat would intensify the experience of self-motion (as explained above, because ANOVA does not compare the dependent variable to zero, rather, it only compares between groups, it could not be used to test the *threat enhancement* hypothesis). Because modality-independent PSEs only inform whether or not there was an overall effect of threat across both modalities (and not how each modality was itself affected), we also analyzed the vestibular and visual PSEs, separately. The *vestibular enhancement* hypothesis was tested using a paired (within-subject) one-tailed *t*-test (in line with the a priori hypothesis that threat would intensify vestibular perception more than visual). The *trait anxiety enhancement* hypothesis was tested (and *vestibular enhancement* results confirmed) using a repeated-measures ANCOVA with modality (visual/vestibular) as the repeated-measures factor and STAI-T scores as a covariate [123, 136]. Each hypothesis was independent of the others. Accordingly, we report the raw *p*-values in the Results.

In post hoc analysis, we discovered that threat actually diminished visual perception of self-motion (contrary to our hypothesis). We still first and foremost report results from one-tailed tests (and only afterwards present two-tailed results to describe this finding) because (i) this more accurately reflects our a priori threat enhancement hypothesis; (ii) it more accurately presents that the visual results did not follow this a priori hypothesis (visual results using the one-tailed test were not significant); and (iii) presenting two-tailed results for the visual condition only afterwards highlights the fact that the latter was post hoc. Lastly, it is worth noting that all the main results in this study that were significant with one-tailed tests maintain their significance when using two-tailed tests.

## Data Availability

All data generated or analyzed during this study are included in this published article and its supplementary information files [137]. Figshare: https://doi.org/10.6084/m9.figshare.25707660.

## Abbreviations

PIVC: Parieto-insular vestibular cortex
VIP: Ventral intraparietal
STAI-T: State-Trait Anxiety Inventory, trait component
HMD: Head-mounted display
IADS: International Affective Digitized Sounds
PSE: Point of subjective equality
MSTd: Dorsal medial superior temporal area

## References

[1] Cole S, Balcetis E, Dunning D (2013). Affective signals of threat increase perceived proximity. Psychol Sci.

[2] Tabor A, Catley MJ, Gandevia SC, Thacker MA, Spence C, Moseley GL (2015). The close proximity of threat: altered distance perception in the anticipation of pain. Front Psychol.

[3] Xiao YJ, van Bavel JJ (2012). See your friends close and your enemies closer: social identity and identity threat shape the representation of physical distance. Personal Soc Psychol Bull.

[4] Vagnoni E, Lourenco SF, Longo MR. Threat modulates perception of looming visual stimuli. Curr Biol. 2012;22(19):R826–7. Available from: 10.1016/j.cub.2012.07.053. 10.1016/j.cub.2012.07.053 23058796

[5] Witt JK, Sugovic M (2013). Spiders appear to move faster than non-threatening objects regardless of one’s ability to block them. Acta Psychol (Amst).

[6] Tajadura-Jiménez A, Väljamäe A, Asutay E, Västfjäll D (2010). Embodied auditory perception: the emotional impact of approaching and receding sound sources. Emotion.

[7] Droit-Volet S, Brunot S, Niedenthal PM (2004). Perception of the duration of emotional events. Cogn Emot.

[8] Noulhiane M, Mella N, Samson S, Ragot R, Pouthas V (2007). How Emotional Auditory Stimuli Modulate Time Perception. Emotion.

[9] van Wassenhove V, Buonomano DV, Shimojo S, Shams L (2008). Distortions of subjective time perception within and across senses. PLoS One.

[10] Bishop SJ (2007). Neurocognitive mechanisms of anxiety: an integrative account. Trends Cogn Sci.

[11] Teachman BA, Stefanucci JK, Clerkin EM, Cody MW, Proffitt DR (2008). A new mode of fear expression: perceptual bias in height fear. Emotion.

[12] Clerkin EM, Cody MW, Stefanucci JK, Proffitt DR, Teachman BA (2009). Imagery and fear influence height perception. J Anxiety Disord.

[13] Cisler JM, Koster EHW. Mechanisms of attentional biases towards threat in anxiety disorders: an integrative review. Clin Psychol Rev. 2010;30(2):203–16. Available from: 10.1016/j.cpr.2009.11.003. 10.1016/j.cpr.2009.11.003 PMC2814889 20005616

[14] Krupić D, Žuro B, Corr PJ (2021). Anxiety and threat magnification in subjective and physiological responses of fear of heights induced by virtual reality. Pers Individ Dif.

[15] Riskind JH (1999). The psychology of looming vulnerability: its relationships to loss. J Pers Interpers Loss.

[16] Riskind JH, Moore R, Bowley L (1995). The looming of spiders: the fearful perceptual distortion of movement and menace. Behav Res Ther.

[17] Riskind JH, Kleiman EM, Seifritz E, Neuhoff J. Influence of anxiety, depression and looming cognitive style on auditory looming perception. J Anxiety Disord. 2014;28(1):45–50. Available from: 10.1016/j.janxdis.2013.11.005. 10.1016/j.janxdis.2013.11.005 24361906

[18] Tipples J (2008). Negative emotionality influences the effects of emotion on time perception. Emotion.

[19] Bar-Haim Y, Kerem A, Lamy D, Zakay D (2010). When time slows down: the influence of threat on time perception in anxiety. Cogn Emot.

[20] Harrison OK, Köchli L, Marino S, Luechinger R, Hennel F, Brand K, et al. Interoception of breathing and its relationship with anxiety. Neuron. 2021;109(24):4080–4093.e8. Available from: http://www.cell.com/article/S0896627321007182/fulltext. Cited 2022 Jan 3. 10.1016/j.neuron.2021.09.045 PMC8691949 34672986

[21] Schmitz J, Blechert J, Krämer M, Asbrand J, Tuschen-Caffier B. Biased perception and interpretation of bodily anxiety symptoms in childhood social anxiety. 101080/153744162012632349. 2012;41(1):92–102. Available from: https://www.tandfonline.com/doi/abs/10.1080/15374416.2012.632349. Cited 2023 Feb 26. 10.1080/15374416.2012.632349 22233249

[22] Stevens S, Gerlach AL, Cludius B, Silkens A, Craske MG, Hermann C (2011). Heartbeat perception in social anxiety before and during speech anticipation. Behav Res Ther.

[23] Smith R, Kuplicki R, Feinstein J, Forthman KL, Stewart JL, Paulus MP, et al. A Bayesian computational model reveals a failure to adapt interoceptive precision estimates across depression, anxiety, eating, and substance use disorders. PLOS Comput Biol. 2020;16(12):e1008484. Available from: https://journals.plos.org/ploscompbiol/article?id=10.1371/journal.pcbi.1008484. Cited 2023 Feb 8. 10.1371/journal.pcbi.1008484 PMC7769623 33315893

[24] Smith R, Feinstein JS, Kuplicki R, Forthman KL, Stewart JL, Paulus MP, et al. Perceptual insensitivity to the modulation of interoceptive signals in depression, anxiety, and substance use disorders. Sci Reports 2021 111 [Internet]. 2021;11(1):1–14. Available from: https://www.nature.com/articles/s41598-021-81307-3. Cited 2023 Feb 8. 10.1038/s41598-021-81307-3 PMC7822872 33483527

[25] Adkin AL, Carpenter MG (2018). New insights on emotional contributions to human postural control. Front Neurol.

[26] Horslen BC, Carpenter MG. Arousal, valence and their relative effects on postural control. Exp Brain Res. 2011;215(1):27–34. Available from: https://link.springer.com/article/10.1007/s00221-011-2867-9. Cited 2023 Jan 31. 10.1007/s00221-011-2867-9 21947171

[27] Zaback M, Luu MJ, Adkin AL, Carpenter MG. Selective preservation of changes to standing balance control despite psychological and autonomic habituation to a postural threat. Sci Reports 2021 111. 2021;11(1):1–15. Available from: https://www.nature.com/articles/s41598-020-79417-5. Cited 2023 Jan 28. 10.1038/s41598-020-79417-5 PMC7801693 33431937

[28] Rzyski K, Nielsen EI, Zaback M, Luu MJ, Carpenter MG. Postural and emotional changes following repeated exposure to standing at a virtual height. J Exerc Movement, Sport (SCAPPS Ref Abstr Repos. 2019;51(1):52–52. Available from: https://www.scapps.org/jems/index.php/1/article/view/2270. Cited 2023 Jan 28.

[29] Johnson KJ, Zaback M, Tokuno CD, Carpenter MG, Adkin AL (2019). Repeated exposure to the threat of perturbation induces emotional, cognitive, and postural adaptations in young and older adults. Exp Gerontol.

[30] Riccelli R, Indovina I, Staab JP, Nigro S, Augimeri A, Lacquaniti F (2017). Neuroticism modulates brain visuo-vestibular and anxiety systems during a virtual rollercoaster task. Hum Brain Mapp.

[31] Dichgans J, Brandt T. Visual-vestibular interaction: effects on self-motion perception and postural control. In: Perception. Springer Berlin Heidelberg; 1978. p. 755–804.

[32] Fushiki H, Kobayashi K, Asai M, Watanabe Y. Influence of visually induced self-motion on postural stability. Acta Otolaryngol. 2005;125(1):60–4. Available from: https://www.tandfonline.com/doi/abs/10.1080/00016480410015794. Cited 2021 Apr 9. 10.1080/00016480410015794 15799576

[33] Gu Y, DeAngelis GC, Angelaki DE. A functional link between area MSTd and heading perception based on vestibular signals. Nat Neurosci. 2007;10(8):1038–47. Available from: /record/2008–05015–018. Cited 2021 Apr 9. 10.1038/nn1935 PMC2430983 17618278

[34] Fetsch CR, Turner AH, DeAngelis GC, Angelaki DE (2009). Dynamic reweighting of visual and vestibular cues during self-motion perception. J Neurosci.

[35] Butler JS, Smith ST, Campos JL, Bülthoff HH. Bayesian integration of visual and vestibular signals for heading. J Vis. 2010;10(11):1–13. Available from: https://pubmed.ncbi.nlm.nih.gov/20884518/. Cited 2021 Apr 9. 10.1167/10.11.23 20884518

[36] Zaidel A, Goin-Kochel RP, Angelaki DE. Self-motion perception in autism is compromised by visual noise but integrated optimally across multiple senses. Proc Natl Acad Sci U S A. 2015;112(20):6461–6. Available from: https://www.pnas.org/content/112/20/6461. Cited 2021 Apr 9. 10.1073/pnas.1506582112 PMC4443344 25941373

[37] Warren WH, Hannon DJ. Direction of self-motion is perceived from optical flow. Nature. 1988;336(6195):162–3. Available from: https://www.nature.com/articles/336162a0. Cited 2021 Jun 22.

[38] Beisel KW, Wang-Lundberg Y, Maklad A, Fritzsch B (2005). Development and evolution of the vestibular sensory apparatus of the mammalian ear. J Vestib Res.

[39] Straka H, Zwergal A, Cullen KE. Vestibular animal models: contributions to understanding physiology and disease. J Neurol. 2016;263(1):10–23. Available from: https://link.springer.com/article/10.1007/s00415-015-7909-y. 10.1007/s00415-015-7909-y PMC4833800 27083880

[40] Ayres AJ. Sensory integration and learning disorders. Los Angeles Calif: Western Psychological Services; 1972

[41] Kaga K. Embryology of inner ear and its malformation. Cochlear Implant Child with Inn Ear Malform Cochlear Nerve Defic. 2017;11–8. Available from: https://link.springer.com/chapter/10.1007/978-981-10-1400-0_2. Cited 2022 Jan 3.

[42] Angelaki DE, Cullen KE. Vestibular system: the many facets of a multimodal sense. Annu Rev Neurosci. 2008;31(1):125–50. Available from: http://www.annualreviews.org/doi/10.1146/annurev.neuro.31.060407.125555. Cited 2021 Feb 8. 10.1146/annurev.neuro.31.060407.125555 18338968

[43] Goldberg JM, Cullen KE. Vestibular control of the head: possible functions of the vestibulocollic reflex. Exp brain Res. 2011;210(0):331. Available from: /pmc/articles/PMC4157641/. Cited 2022 Jan 3. 10.1007/s00221-011-2611-5 PMC4157641 21442224

[44] Peusner KD, Shao M, Reddaway R, Hirsch JC (2012). Basic concepts in understanding recovery of function in vestibular reflex networks during vestibular compensation. Front Neurol.

[45] Wilson VJ, Boyle R, Fukushima K, Rose PK, Shinoda Y, Sugiuchi Y (1995). The vestibulocollic reflex. J Vestib Res.

[46] Balaban CD, Thayer JF (2001). Neurological bases for balance-anxiety links. J Anxiety Disord.

[47] Jacob RG, Furman JM. Psychiatric consequences of vestibular dysfunction. Curr Opin Neurol. 2001;14(1):41–6. Available from: http://www.ncbi.nlm.nih.gov/pubmed/11176216. Cited 2019 Nov 26. 10.1097/00019052-200102000-00007 11176216

[48] Stein MB, Asmundson GJG, Ireland D, Walker JR (1994). Panic disorder in patients attending a clinic for vestibular disorders. Am J Psychiatry.

[49] Asmundson GJG, Larsen DK, Stein MB (1998). Panic disorder and vestibular disturbance: an overview of empirical findings and clinical implications. J Psychosom Res.

[50] Jacob RG, Furman JM, Durrant JD, Turner SM (1996). Panic, agoraphobia, and vestibular dysfunction. Am J Psychiatry.

[51] Eagger S, Luxon LM, Davies RA, Coelho A, Ron MA (1992). Psychiatric morbidity in patients with peripheral vestibular disorder: a clinical and neuro-otological study. J Neurol Neurosurg Psychiatry.

[52] Simon NM, Pollack MH, Tuby KS, Stern TA (1998). Dizziness and panic disorder: a review of the association between vestibular dysfunction and anxiety. Ann Clin Psychiatry.

[53] Nagaratnam N, Ip J, Bou-Haidar P (2005). The vestibular dysfunction and anxiety disorder interface: a descriptive study with special reference to the elderly. Arch Gerontol Geriatr.

[54] Levinson HN. The cerebellar-vestibular predisposition to anxiety disorders. Percept Mot Skills. 1989;68(1):323–38. Available from: http://www.ncbi.nlm.nih.gov/pubmed/2928066. Cited 2020 Jan 28. 10.2466/pms.1989.68.1.323 2928066

[55] Toupet M, Van Nechel C, Hautefort C, Heuschen S, Duquesne U, Cassoulet A, et al. Influence of visual and vestibular hypersensitivity on derealization and depersonalization in chronic dizziness. Front Neurol . 2019;10. Available from: https://www.frontiersin.org/article/10.3389/fneur.2019.00069/full. Cited 2019 Nov 26. 10.3389/fneur.2019.00069 PMC6381029 30814972

[56] Staab JP, Rohe DE, Eggers SDZ, Shepard NT. Anxious, introverted personality traits in patients with chronic subjective dizziness. J Psychosom Res. 2014;76(1):80–3. Available from: https://pubmed.ncbi.nlm.nih.gov/24360146/. Cited 2021 Feb 9. 10.1016/j.jpsychores.2013.11.008 24360146

[57] Hainaut JP, Caillet G, Lestienne FG, Bolmont B (2011). The role of trait anxiety on static balance performance in control and anxiogenic situations. Gait Posture.

[58] Coelho CM, Wallis G. Deconstructing acrophobia: physiological and psychological precursors to developing a fear of heights. Depress Anxiety. 2010;27(9):864–70. Available from: http://doi.wiley.com/10.1002/da.20698. Cited 2021 Feb 8. 10.1002/da.20698 20821801

[59] Hüweler R, Kandil FI, Alpers GW, Gerlach AL (2009). The impact of visual flow stimulation on anxiety, dizziness, and body sway in individuals with and without fear of heights. Behav Res Ther.

[60] Jacob RG, Redfern MS, Furman JM (1995). Optic flow-induced sway in anxiety disorders associated with space and motion discomfort. J Anxiety Disord.

[61] Redfern MS, Yardley L, Bronstein AM. Visual influences on balance. J Anxiety Disord. 2001;15(1–2):81–94. Available from: https://pubmed.ncbi.nlm.nih.gov/11388359/. Cited 2021 Feb 9. 10.1016/s0887-6185(00)00043-8 11388359

[62] Coelho CM, Balaban CD. Visuo-vestibular contributions to anxiety and fear. Neurosci Biobehav Rev. 2015;48:148–59. Available from: 10.1016/j.neubiorev.2014.10.023. 25451199

[63] Viaud-Delmon I, Ivanenko YP, Berthoz A, Jouvent R (2000). Adaptation as a sensorial profile in trait anxiety: a study with virtual reality. J Anxiety Disord.

[64] Warren WH. Optic flow. In: The Senses: A Comprehensive Reference. Elsevier Inc.; 2008. p. 219–30.

[65] Brandt T, Dichgans J, Koenig E (1973). Differential effects of central verses peripheral vision on egocentric and exocentric motion perception. Exp brain Res.

[66] Berthoz A, Pavard B, Young LR. Perception of linear horizontal self-motion induced by peripheral vision (linearvection) basic characteristics and visual-vestibular interactions. Exp Brain Res. 1975;23(5):471–89. Available from: https://link.springer.com/article/10.1007/BF00234916. Cited 2021 Apr 9. 10.1007/BF00234916 1081949

[67] Warren WH, Saunders JA (1995). Perceiving heading in the presence of moving objects. Perception.

[68] Royden CS, Banks MS, Crowell JA (1992). The perception of heading during eye movements. Nature.

[69] Lappe M, Bremmer F, Van Den Berg AV (1999). Perception of self-motion from visual flow. Trends Cogn Sci.

[70] Dokka K, Park H, Jansen M, DeAngelis GC, Angelaki DE. Causal inference accounts for heading perception in the presence of object motion. Proc Natl Acad Sci U S A. 2019;116(18):9060–5. Available from: https://www.pnas.org/content/116/18/9060. Cited 2021 Nov 10. 10.1073/pnas.1820373116 PMC6500172 30996126

[71] Sasaki R, Angelaki DE, DeAngelis GC. Dissociation of self-motion and object motion by linear population decoding that approximates marginalization. J Neurosci. 2017;37(46):11204–19. Available from: https://www.jneurosci.org/content/37/46/11204. Cited 2021 Nov 10. 10.1523/JNEUROSCI.1177-17.2017 PMC5688528 29030435

[72] Seno T, Ito H, Sunaga S (2010). Vection aftereffects from expanding/contracting stimuli. Seeing Perceiving.

[73] Bubka A, Bonato F, Palmisano S. Expanding and contracting optic-flow patterns and vection. Perception. 2008;37(5):704–11. Available from: http://www.ncbi.nlm.nih.gov/pubmed/18605144. Cited 2020 Jan 27. 10.1068/p5781 18605144

[74] Chen A, DeAngelis GC, Angelaki DE. Macaque parieto-insular vestibular cortex: responses to self-motion and optic flow. J Neurosci. 2010;30(8):3022–42. Available from: https://pubmed.ncbi.nlm.nih.gov/20181599/. Cited 2023 Feb 19. 10.1523/JNEUROSCI.4029-09.2010 PMC3108058 20181599

[75] Avillac M, Denève S, Olivier E, Pouget A, Duhamel JR. Reference frames for representing visual and tactile locations in parietal cortex. Nat Neurosci. 2005;8(7):941–9. Available from: https://pubmed.ncbi.nlm.nih.gov/15951810/. Cited 2023 Mar 10. 10.1038/nn1480 15951810

[76] Sereno MI, Huang RS (2014). Multisensory maps in parietal cortex. Curr Opin Neurobiol.

[77] Zeng F, Zaidel A, Chen A. Contrary neuronal recalibration in different multisensory cortical areas. Elife. 2023;12. Available from: https://elifesciences.org/articles/82895. Cited 2023 Mar 10. 10.7554/eLife.82895 PMC9988259 36877555

[78] Auricchio A, Stellbrink C, Block M, Sack S, Vogt J, Bakker P, et al. Effect of pacing chamber and atrioventricular delay on acute systolic function of paced patients with congestive heart failure. Circulation. 1999;99(23):2993–3001. Available from: https://www.ahajournals.org/doi/abs/10.1161/01.CIR.99.23.2993. Cited 2021 Nov 9. 10.1161/01.cir.99.23.2993 10368116

[79] Jürgens R, Becker W. Perception of angular displacement without landmarks: evidence for Bayesian fusion of vestibular, optokinetic, podokinesthetic, and cognitive information. Exp Brain Res 2006 1743. 2006;174(3):528–43. Available from: https://link.springer.com/article/10.1007/s00221-006-0486-7. Cited 2021 Nov 9. 10.1007/s00221-006-0486-7 16832684

[80] Campos JL, Butler JS, Bülthoff HH. Contributions of visual and proprioceptive information to travelled distance estimation during changing sensory congruencies. Exp Brain Res 2014 23210 [Internet]. 2014;232(10):3277–89. Available from: https://link.springer.com/article/10.1007/s00221-014-4011-0. Cited 2021 Nov 9. 10.1007/s00221-014-4011-0 24961739

[81] Seno T, Murata K, Fujii Y, Kanaya H, Ogawa M, Tokunaga K, et al. Vection is enhanced by increased exposure to optic flow. Iperception. 2018;9(3):2041669518774069. Available from: http://www.ncbi.nlm.nih.gov/pubmed/30046430. Cited 2021 Nov 14. 10.1177/2041669518774069 PMC6055108 30046430

[82] Guldin WO, Grüsser OJ (1998). Is there a vestibular cortex?. Trends Neurosci.

[83] Suzuki M, Kitano H, Ito R, Kitanishi T, Yazawa Y, Ogawa T (2001). Cortical and subcortical vestibular response to caloric stimulation detected by functional magnetic resonance imaging. Cogn Brain Res.

[84] Lopez C, Blanke O, Mast FW. The human vestibular cortex revealed by coordinate-based activation likelihood estimation meta-analysis. Neuroscience. 2012;212:159–79. Available from: https://pubmed.ncbi.nlm.nih.gov/22516007/. Cited 2021 Nov 11. 10.1016/j.neuroscience.2012.03.028 22516007

[85] Zu Eulenburg P, Caspers S, Roski C, Eickhoff SB (2012). Meta-analytical definition and functional connectivity of the human vestibular cortex. Neuroimage.

[86] Mufson EJ, Mesulam MM, Pandya DN. Insular interconnections with the amygdala in the rhesus monkey. Neuroscience. 1981;6(7):1231–48. Available from: https://pubmed.ncbi.nlm.nih.gov/6167896/. Cited 2023 Feb 22. 10.1016/0306-4522(81)90184-6 6167896

[87] Almashaikhi T, Rheims S, Ostrowsky-Coste K, Montavont A, Jung J, De Bellescize J, et al. Intrainsular functional connectivity in human. Hum Brain Mapp. 2014;35(6):2779. Available from: /pmc/articles/PMC6869822/. Cited 2023 Feb 22. 10.1002/hbm.22366 PMC6869822 24027207

[88] Almashaikhi T, Rheims S, Jung J, Ostrowsky-Coste K, Montavont A, De Bellescize J, et al. Functional connectivity of insular efferences. Hum Brain Mapp. 2014;35:10. Available from: https://onlinelibrary.wiley.com/doi/10.1002/hbm.22549. Cited 2023 Feb 22. 10.1002/hbm.22549 PMC6869741 24839121

[89] Riccelli R, Passamonti L, Toschi N, Nigro S, Chiarella G, Petrolo C, et al. Altered insular and occipital responses to simulated vertical self-motion in patients with persistent postural-perceptual dizziness. Front Neurol. 2017;8(OCT):529. 10.3389/fneur.2017.00529 PMC5650964 29089920

[90] Balaban CD (2004). Projections from the parabrachial nucleus to the vestibular nuclei: potential substrates for autonomic and limbic influences on vestibular responses. Brain Res.

[91] Petrovich GD, Swanson LW. Projections from the lateral part of the central amygdalar nucleus to the postulated fear conditioning circuit. Brain Res. 1997;763(2):247–54. Available from: https://pubmed.ncbi.nlm.nih.gov/9296566/. Cited 2021 Feb 12. 10.1016/s0006-8993(96)01361-3 9296566

[92] Carmona JE, Holland AK, Harrison DW. Extending the functional cerebral systems theory of emotion to the vestibular modality: a systematic and integrative approach. Psychol Bull. 2009;135(2):286–302. Available from: https://pubmed.ncbi.nlm.nih.gov/19254081/. Cited 2021 Feb 9. 10.1037/a0014825 19254081

[93] Indovina I, Riccelli R, Staab JP, Lacquaniti F, Passamonti L (2014). Personality traits modulate subcortical and cortical vestibular and anxiety responses to sound-evoked otolithic receptor stimulation. J Psychosom Res.

[94] Everhart DE, Harrison DW. Heart rate and fluency performance among high- and low-anxious men following autonomic stress. Int J Neurosci. 2002;112(10):1149–71. Available from: https://pubmed.ncbi.nlm.nih.gov/12587519/. Cited 2021 Feb 12. 10.1080/00207450290026120 12587519

[95] Angelaki DE. How optic flow and inertial cues improve motion perception. Cold Spring Harb Symp Quant Biol. 2014;79:141–8. Available from: http://symposium.cshlp.org/content/79/141.full. Cited 2021 Nov 10. 10.1101/sqb.2014.79.024638 26092884

[96] Royden CS, Hildreth EC (1996). Human heading judgments in the presence of moving objects. Percept Psychophys.

[97] Logan DJ, Duffy CJ. Cortical area MSTd combines visual cues to represent 3-D self-movement. Cereb Cortex. 2006;16(10):1494–507. Available from: https://academic.oup.com/cercor/article/16/10/1494/353925. Cited 2021 Nov 14. 10.1093/cercor/bhj082 16339087

[98] Gu Y, deAngelis GC, Angelaki DE. Causal links between dorsal medial superior temporal area neurons and multisensory heading perception. J Neurosci. 2012;32(7):2299–313. Available from: https://www.jneurosci.org/content/32/7/2299. Cited 2021 Nov 14. 10.1523/JNEUROSCI.5154-11.2012 PMC3311305 22396405

[99] Britten KH, Van Wezel RJA. Area MST and heading perception in macaque monkeys. Cereb Cortex. 2002;12(7):692–701. Available from: https://pubmed.ncbi.nlm.nih.gov/12050081/. Cited 2021 Nov 14. 10.1093/cercor/12.7.692 12050081

[100] Britten KH, Van Wezel RJA. Electrical microstimulation of cortical area MST biases heading perception in monkeys. Nat Neurosci 1998 11. 1998;1(1):59–63. Available from: https://www.nature.com/articles/nn0598_59. Cited 2021 Nov 14. 10.1038/259 10195110

[101] Li W, Lu J, Zhu Z, Gu Y. Causal contribution of optic flow signal in Macaque extrastriate visual cortex for roll perception. Nat Commun 2022 131. 2022;13(1):1–17. Available from: https://www.nature.com/articles/s41467-022-33245-5. Cited 2023 Mar 11. 10.1038/s41467-022-33245-5 PMC9485245 36123363

[102] Yu X, Gu Y. Probing sensory readout via combined choice-correlation measures and microstimulation perturbation. Neuron. 2018;100(3):715–727.e5. Available from: https://pubmed.ncbi.nlm.nih.gov/30244884/. Cited 2023 Mar 11. 10.1016/j.neuron.2018.08.034 30244884

[103] Malcolm BR, Foxe JJ, Butler JS, Molholm S, De Sanctis P. Cognitive load reduces the effects of optic flow on gait and electrocortical dynamics during treadmill walking. J Neurophysiol. 2018;120(5):2246–59. Available from: https://journals.physiology.org/doi/10.1152/jn.00079.2018. Cited 2023 Feb 20. 10.1152/jn.00079.2018 PMC6295527 30067106

[104] Rancz EA, Moya J, Drawitsch F, Brichta AM, Canals S, Margrie TW. Widespread vestibular activation of the rodent cortex. J Neurosci. 2015;35(15):5926–34. Available from: https://www.jneurosci.org/content/35/15/5926. Cited 2021 Apr 9. 10.1523/JNEUROSCI.1869-14.2015 PMC4397593 25878265

[105] Glasauer S, Schneider E, Grasso R, Ivanenko YP. Space-time relativity in self-motion reproduction. J Neurophysiol. 2007;97(1):451–61. Available from: https://journals.physiology.org/doi/10.1152/jn.01243.2005. Cited 2023 Feb 25. 10.1152/jn.01243.2005 17050823

[106] Buhusi CV, Meck WH (2005). What makes us tick? Functional and neural mechanisms of interval timing. Nat Rev Neurosci.

[107] Droit-Volet S, Meck WH (2007). How emotions colour our perception of time. Trends Cogn Sci.

[108] Wearden JH, Penton-Voak IS. Feeling the heat: body temperature and the rate of subjective time, revisited. Q J Exp Psychol B. 1995;48(2):129–41. Available from: http://www.ncbi.nlm.nih.gov/pubmed/7597195. Cited 2020 Jan 26. 10.1080/14640749508401443 7597195

[109] Fortin C. Attentional time-sharing in interval timing. In: Functional and Neural Mechanisms of Interval Timing. CRC Press; 2003. p. 235–60.

[110] Buhusi CV, Meck WH (2006). Interval timing with gaps and distracters: evaluation of the ambiguity, switch, and time-sharing hypotheses. J Exp Psychol Anim Behav Process.

[111] Lake JI (2016). Recent advances in understanding emotion-driven temporal distortions. Curr Opin Behav Sci.

[112] Block RA, Hancock PA, Zakay D (2010). How cognitive load affects duration judgments: a meta-analytic review. Acta Psychol (Amst).

[113] Price JL (2003). Comparative aspects of amygdala connectivity. Ann N Y Acad Sci.

[114] Amaral DG, Price JL. Amygdalo-cortical projections in the monkey (Macaca fascicularis). J Comp Neurol. 1984;230(4):465–96. Available from: https://onlinelibrary.wiley.com/doi/full/10.1002/cne.902300402. Cited 2021 Nov 16. 10.1002/cne.902300402 6520247

[115] Iwai E, Yukie M. Amygdalofugal and amygdalopetal connections with modality-specific visual cortical areas in macaques (macaca fuscata, M. mulatta, and M. fascicularis). J Comp Neurol. 1987;261(3):362–87. Available from: https://onlinelibrary.wiley.com/doi/full/10.1002/cne.902610304. Cited 2021 Nov 16. 10.1002/cne.902610304 3611417

[116] Balaban CD. Neural substrates linking balance control and anxiety. Physiol Behav. 2002;77(4–5):469–75. Available from: https://pubmed.ncbi.nlm.nih.gov/12526985/. Cited 2021 Feb 9. 10.1016/s0031-9384(02)00935-6 12526985

[117] Chen YC, Chen C, Martínez RM, Fan YT, Liu CC, Chen CY, et al. An amygdala-centered hyper-connectivity signature of threatening face processing predicts anxiety in youths with autism spectrum conditions. Autism Res. 2021;14(11):2287–99. Available from: https://onlinelibrary.wiley.com/doi/full/10.1002/aur.2595. Cited 2022 Jan 11. 10.1002/aur.2595 34423915

[118] Donnici C, Long X, Dewey D, Letourneau N, Landman B, Huo Y, et al. Prenatal and postnatal maternal anxiety and amygdala structure and function in young children. Sci Reports 2021 111. 2021;11(1):1–12. Available from: https://www.nature.com/articles/s41598-021-83249-2. Cited 2022 Jan 11. 10.1038/s41598-021-83249-2 PMC7889894 33597557

[119] Gibbard CR, Ren J, Skuse DH, Clayden JD, Clark CA. Structural connectivity of the amygdala in young adults with autism spectrum disorder. Hum Brain Mapp. 2018;39(3):1270–82. Available from: https://onlinelibrary.wiley.com/doi/full/10.1002/hbm.23915. Cited 2022 Jan 11. 10.1002/hbm.23915 PMC5838552 29265723

[120] Kuppens P, Tuerlinckx F, Russell JA, Barrett LF (2013). The relation between valence and arousal in subjective experience. Psychol Bull.

[121] Campos J, Ramkhalawansingh R, Pichora-Fuller MK. Hearing, self-motion perception, mobility, and aging. Hear Res. 2018;369:42–55. Available from: https://pubmed.ncbi.nlm.nih.gov/29661612/. Cited 2023 Mar 11. 10.1016/j.heares.2018.03.025 29661612

[122] Di Lernia D, Serino S, Pezzulo G, Pedroli E, Cipresso P, Riva G (2018). Feel the time. Time perception as a function of interoceptive processing. Front Hum Neurosci.

[123] Tipples J (2011). When time stands still: fear-specific modulation of temporal bias due to threat. Emotion.

[124] Spielberger CD, Gorsuch RL. State-trait Anxiety Inventory for Adults: Manual and Sample: Manual, Instrument and Scoring Guide, vol 4. Palo Alto: Consulting Psychologists Press; 1983. p. 0–78

[125] Mathews A, Mackintosh B. Take a closer look: emotion modifies the boundary extension effect. Emotion. 2004;4(1):36–45. Available from: http://www.ncbi.nlm.nih.gov/pubmed/15053725. Cited 2020 Feb 23. 10.1037/1528-3542.4.1.36 15053725

[126] Perkins AM, Cooper A, Abdelall M, Smillie LD, Corr PJ (2010). Personality and defensive reactions: fear, trait anxiety, and threat magnification. J Pers.

[127] Viaud-Delmon I, Berthoz A, Jouvent R (2002). Multisensory integration for spatial orientation in trait anxiety subjects: absence of visual dependence. Eur Psychiatry.

[128] Tipples J (2019). Increased temporal sensitivity for threat: a Bayesian generalized linear mixed modeling approach. Attention, Perception, Psychophys.

[129] Gu Y, Angelaki DE, DeAngelis GC (2008). Neural correlates of multisensory cue integration in macaque MSTd. Nat Neurosci.

[130] Bradley MM, Lang PJ. The International Affective Digitized Sounds (IADS-2): Affective ratings of sounds and instruction manual. Technical report B-3, vol 434. Gainesville: University of Florida; 2007. p. 30.

[131] Yang W, Makita K, Nakao T, Kanayama N, Machizawa MG, Sasaoka T (2018). Affective auditory stimulus database: an expanded version of the International Affective Digitized Sounds (IADS-E). Behav Res Methods.

[132] Cornsweet TN (1962). The staircrase-method in psychophysics. Am J Psychol.

[133] Leek MR. Adaptive procedures in psychophysical research. Percept Psychophys. 2001;63(8):1279–92. Available from: https://link.springer.com/article/10.3758/BF03194543. Cited 2021 Feb 14. 10.3758/bf03194543 11800457

[134] Schütt HH, Harmeling S, Macke JH, Wichmann FA. Painfree and accurate Bayesian estimation of psychometric functions for (potentially) overdispersed data. Vision Res. 2016;122:105–23. Available from: https://pubmed.ncbi.nlm.nih.gov/27013261/. Cited 2021 Jan 17. 10.1016/j.visres.2016.02.002 27013261

[135] Hosmer DW, Lemeshow S (1989). Applied logistic regression.

[136] Indovina I, Robbins TW, Núñez-Elizalde AO, Dunn BD, Bishop SJ (2011). Fear-conditioning mechanisms associated with trait vulnerability to anxiety in humans. Neuron.

[137] Hacohen-Brown S, Gilboa-Schechtman E, Zaidel A. Modality-specific effects of threat on self-motion perception. figshare. 10.6084/m9.figshare.25707660. 10.1186/s12915-024-01911-3 PMC11119305 38783286

